# Quantitative Detection of miRNA-21 Expression in Tumor Cells and Tissues Based on Molecular Beacon

**DOI:** 10.1155/2018/3625823

**Published:** 2018-09-02

**Authors:** Qingxin Liu, Jialong Fan, Chuang Zhou, Liqun Wang, Bin Zhao, Haibin Zhang, Bin Liu, Chunyi Tong

**Affiliations:** ^1^College of Veterinary Medicine, Nanjing Agricultural University, Nanjing, Jiangsu 210095, China; ^2^Jiangsu Vocational College of Agriculture and Forestry, Jurong, Jiangsu 212400, China; ^3^College of Biology, Hunan University, Changsha, Hunan 410082, China

## Abstract

As a new tumor marker, the microRNA-21 (miRNA21) level can provide important information for early diagnosis, drug treatment, and prognosis of gastric cancer. With the tool of molecular beacons which can hybridize specifically with target miRNA-21 and generate fluorescence signal change, this paper develops a direct, simple, and rapid method for miRNA-21 detection with detection limit of 0.5 nM. Under the optimal conditions, the method was used to detect the expression of miRNA-21 in tumor cells and tissues. The results showed significant differences of miRNA-21 levels in tumor cells which have different origins and different degree of malignancy. In 8 cases of gastric cancer tissues and adjacent tissues, the level of miRNA-21 in 6 cases was higher than that in adjacent tissues, 1 case had lower expression level than that in adjacent tissues, and 1 case had no significant difference. Furthermore, qRT-PCR method was used to verify the detection results based on the fluorescent probe detection method. The consistent results show that the molecular beacon assay has a good prospect in direct and rapid detection of miRNA-21 expression and will be widely used in the functional research and clinical diagnosis of microRNA.

## 1. Introduction

Since Lee et al. [1] first reported on microRNA (miRNA) in 1993, research on miRNA has quickly become a hot research topic in various fields. MiRNAs are a class of small RNA with about 18-25 nucleotides of endogenous noncoding whose expression level changes directly affect cell proliferation, differentiation, development, and apoptosis in many biological processes and regulate more than a third of the gene expression of body [2]. Clinical study results indicate that, due to specific expression in tissues and cells, miRNA not only is directly involved in the development and progression of tumors, but also can be served as noninvasive markers in the early diagnosis and treatment of a tumor [3–11]. Located in the 17q23.2, the coding gene miRNA-21 belongs to the coding region of the vacuole membrane protein gene (VMP1), and is the tenth intron of the VMP1 gene. A large number of studies show that miRNA-21 in tumor tissues and cells is abnormally high [12, 13] and the expression level directly reflects the degree of tumor malignance [14, 15]. Therefore, the detection of miRNA-21 has significant theoretical and practical value in the early diagnosis, treatment, and prognosis evaluation of clinical tumor. At present, methods that can be used for miRNA detection are microarray [16], northern blotting [17], qRT-PCR [18], etc. However, in varying degrees, these methods have disadvantages such as high cost, being time-consuming, having large demand of sample size, and fussy process; in addition, miRNA is very short and its characteristics of high sequence similarity between members of the same family make it difficult to achieve fast, simple, and good reproducibility of miRNA detection. Since the molecular beacon probe has the advantages of strong specificity, short hybridization time, and easy operation [19–22], it is expected to be applied in the miRNA rapid detection and analysis.

## 2. Experimental

### 2.1. Materials and Instruments

Instruments were Hitachi F-2500 fluorescence spectrophotometer (Japan), Thermo Newslab water bath (USA), Bio-Rad fluorescence quantitative PCR (USA), and Thermo trace UV spectrophotometer (USA). All sequences of RNA (miRNA-21 simply “miR21” for short; miRNA-21 with 3 mutation bases simply “miR21m3” for short) and probes (miRNA-21 molecular beacon simply “miR21-MB” for short) were synthesized by Takara and shown in Table 1. Other reagents such as HRP (Promega), TRIzol (Life Technology), miRNA qPCR kit, and DNaseI (Promega) were used. 8 specimens of gastric cancer (cancerous tissues and adjacent tissues) were collected from the Department of Pathology, Chenzhou First People's Hospital, Hunan Province, including 6 males and 2 females, aged between 44 and 74 years old, with an average age of 58 ± 10 years. All pathological specimens were confirmed by pathological diagnosis, including 1 case of high differentiation, 2 cases of middle differentiation, and 5 cases of low differentiation. The tissue samples were stored at −80°C after liquid nitrogen treatment.

### 2.2. Fluorescence Measurement

A final concentration of 100 nM of MiR21-MB was added to total volume of 100 *μ*L standard buffer solution; then the fluorescence intensity was measured using the scanning time and wavelength scanning at the indicated temperature (37°C, *λ*ex = 521 nm, *λ*em = 578 nm). Both of excitation and emission slit widths were set at 10 nm. The background intensity of solution was monitored until it kept stable at the test temperature. Total RNA was then added and the subsequent change of fluorescence intensity was recorded. The emission spectra were measured by exciting samples at 521 nm and scanning the emission between 550 and 650 nm. Fluorescence emission peaks were measured at 578 nm.

### 2.3. Construction of Detection Standard Curve

The solutions containing 100 nM MiR21-MB and different concentrations of miR21 were incubated in PCR amplifier for 3 h at 37°C. Fluorescence emission spectra were recorded as in Section 2.2. Only the fluorescence data at 578 nm (the maximum emission of TAMRA) were used for analysis. Each sample was incubated at 37°C for 10 min before detection to obtain a steady status of fluorescence and detected three times.

### 2.4. Cell Culture and Extraction of Total RNA from Cells and Tissues

Cancer cell lines, Huh7, HCC1102, and HCC20986, were cultured in Dulbecco's Modified Eagle Medium (DMEM, HyClone), supplemented with 10% heat-inactivated fetal bovine serum ((FBS, Gibco) and 1% Antibiotics (Gibco), in a standard incubator (5% CO_2_ atmosphere at 37°C). All glassware was soaked for 12 h with 0.1% DEPC water solution and then sterilized for 30 min by 120°C. Celsius plastic containers are disposable non-RNase products. Add 1 mL TRIzol reagents for 1 glassware and then pipette the cells up and down several times. Shake tubes vigorously by hand for 15 s and incubate them at room temperature for 5 min. Centrifuge the samples at 12,000×g for 15 min at 4°C. Transfer the aqueous phase to a clean tube, add 500 *μ*L of isopropyl alcohol, incubate samples at room temperature for 10 min, and centrifuge at 12,000×g for 10 min at 4°C. Remove the supernatant, add 1 mL of 75% ethanol washing the RNA pellet, mix the sample by vortexing, and centrifuge at 7,500×g for 5 min at 4°C. Remove the supernatant, air-dry the RNA pellet, dissolve RNA in RNase-free water by passing the solution a few times through a pipette tip, and incubate for 10 min at 60°C. Take 1 *μ*L of samples in Nanodrop 2000/2000C spectrophotometer determination of total RNA concentration and 1% agarose gel electrophoresis to verify the integrity of total RNA, stored at −80°C.

### 2.5. The Specificity of Molecular Beacon Detection

Prepare 15% polyacrylamide gel by adding 3 mL ddH_2_O, 5 mL 30% centrifugal tube, acrylamide, 2 mL×5 TBE, 70 *μ*L 10% ammonium persulfate, and 4 mL TEMED in centrifuge tube, mixing them quickly then pouring glue. Electrophoresis is at 100 V for 1 h. Fixing: flush again with ddH_2_O, pour 20 mL fixative, and slightly shake it on the shaker for 10 min. Rinse: wash two times with ddH_2_O, 10 s each time. Add 20 mL staining solution and slightly shake it for 10 min so as to dye it; wash it with ddH_2_O for 10 s, add 30 mL precooling developer, and then after developing it for 5 min, fix it with fixing solution for another 5 min and take pictures for observation.

### 2.6. Quantitative Detection of miRNA-21

The solution containing 3 *μ*g total RNA with DNase I digestion was heated to 95°C for 5 min and then hybridized with 1 *μ*L of MiR21-MB (10 *μ*M) to 100 *μ*L. Fluorescence changes of solutions were detected on FL-2500 fluorometer after incubation at 37°C for 3 h. Record samples when fluorescence signal is at 578 nm, and then calculate the concentration of miRNA-21 based on established standard curve so as to express it with mol•*μ*g^−1^ total RNA.

### 2.7. Quantitative Detection of miRNA-21 by qRT-PCR

cDNA was generated from 3 *μ*g total RNA of cell or tissue with DNase I digestion using reverse transcriptase kit. Each 20 *μ*L reaction solution contained 10 *μ*L 2×miRNA Reaction Buffer Mix, 2 *μ*L 0.1% BSA, 2 *μ*L miRNA PrimeScript RT Enzyme Mix, 1 *μ*L total RNA, and 5 *μ*L RNase-Free H_2_O; qRT-PCR was carried out for Initial activation of 95°C for 10 s, followed by 40 cycles of 95°C for 5 s, 60°C for 20 s. Each 25 *μ*L reaction solution contained 12.5 *μ*L SYBR Premix Ex Taq (2×), 1 *μ*L Universal-RNU6B-Primer (10 *μ*M) or 1 *μ*L has-miR-21-Primer (10 *μ*M), 2 *μ*L cDNA solution, and 9.5 *μ*L ddH_2_O.

## 3. Results and Discussion

### 3.1. Effects of Temperature and Mg^*2*+^ on the Stability of Molecular Beacon

**Figure 1:**
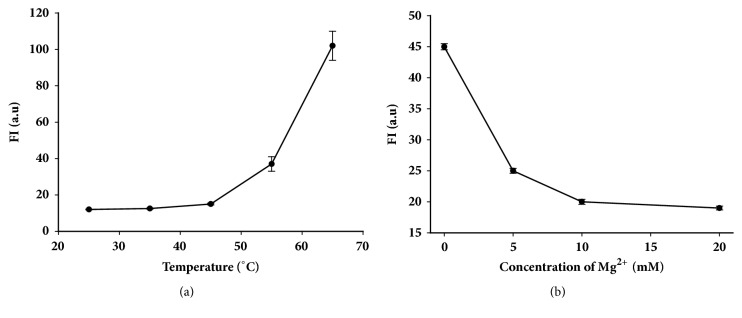
The effects of temperature (a) and Mg^2+^ concentration (b) on the stability of MiR21-MB.

Since temperature and Mg^2+^ can affect the stability of molecular beacon [23–25], in order to select the best conditions to maintain the stability of molecular beacon, we investigated the effect of these two factors. As shown in Figure 1(a), at temperatures below 45°C, fluorescence intensity does not change with increasing temperature, which indicates that molecular beacon can maintain a stable stem-loop structure. When temperature is higher than 45°C, the fluorescence intensity increases slowly with increasing temperature, which indicates that the stem-loop structure of molecular beacon is affected to a certain extent.  Figure 1(b) shows that Mg^2+^ plays an important role in maintaining the stability of the molecular beacon. When the concentration of Mg^2+^ is about 10 mM, the fluorescence background signal is the lowest. In order to ensure the stability and repeatability of the experimental results, the reaction temperature was 45°C. And the Mg^2+^ will affect the hybridization between miR21-MB and miR21, so next the effects will be investigated.

### 3.2. The Effect of pH and Mg^*2*+^ on the Hybridization between miR21-MB and miR21

**Figure 2:**
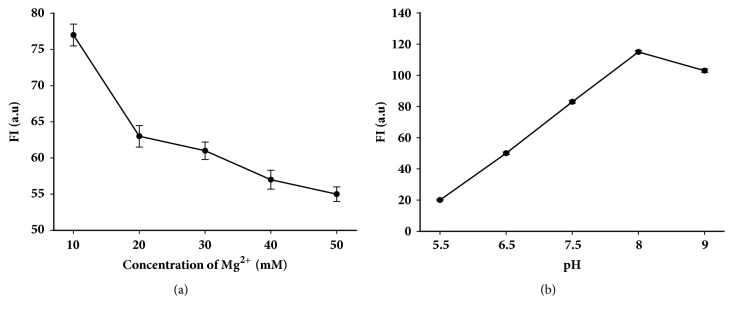
Effects of Mg^2+^ (a) and pH value (b) on the hybridization between miR21-MB and miR21.

We further investigated the influence of Mg^2+^ and pH on the hybridization between miR21-MB and miR21.  Figure 2(a) shows that Mg^2+^ also affects the hybridization process of molecular beacon and miR21. The fluorescence intensity of hybridization process is concentration dependence, and it is pink when Mg^2+^ concentration is 10 mM. The results indicate that more Mg^2+^ may maintain a more stable hairpin structure to decrease the fluorescence signal. Therefore, it is concluded that 10 mM is optimal concentration of Mg^2+^. In addition, Figure 2(b) shows that, at a certain range (5.5-8.0), the fluorescence intensity increases with the increase of pH value, and then the fluorescence signal starts to decrease when the pH value continues to increase. As a result, it is concluded that pH value of 8 is the best condition of pH.

### 3.3. Specificity of Molecular Beacon

**Figure 3:**
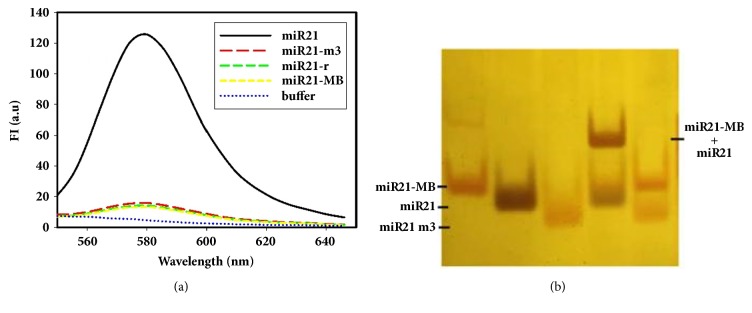
The specificity assay of MB with target by fluorescence spectrometry (a) and polyacrylamide gel electrophoresis staining by silver (b).

The specificity of the hybridization between miR21-MB and target molecules was further investigated by using 3 kinds of miR21 chains including those in random sequence (miR21-r), those with 3 mismatched bases (miR21-m3), and those with complete matching bases (miR21). The fluorescence wavelength scanning results (Figure 3(a)) showed that the fluorescence intensity of miR21-m3 and miR21-r were similar to that of the background, indicating that miR21-MB could not be hybridized with random RNA sequences or mismatched RNA sequences, and the stem cannot be opened to produce fluorescence. But miR21-MB can be opened by the sequence of the complete match of the miR21 hybrid and then produced a significant fluorescence signal. This result indicates that the molecular beacon can distinguish the RNA molecule with different base composition, showing good specificity. We also analyzed the results of miR21-MB/miR21 hybridization using polypropylene gel electrophoresis to verify the results of fluorescence experiments. As shown in Figure 3(b), miR21 and MiR21-MB hybridize and produce hybrid product of new bands in lane 4. Hybridization of miR21-m3 and miR21-MB did not produce new bands in lane 5. These results indicate the strong specificity of the molecular beacon to miR21.

### 3.4. The Detection Standard Curve

**Figure 4:**
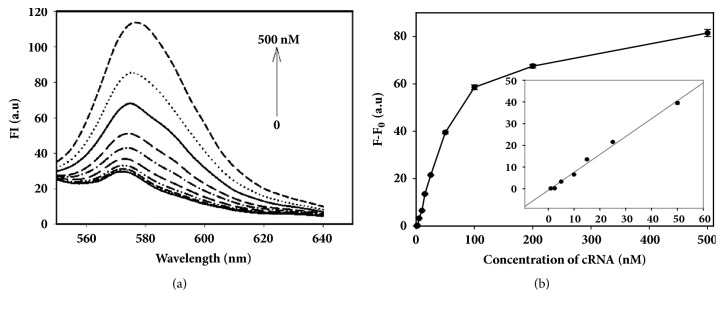
The fluorescence emission spectra of miR21-MB with miR21 (a) and the detection standard curve (b).

The miR21 was prepared by artificial synthesis in vitro. 100 nM miR21-MB was mixed with miR21 with different final concentrations, respectively (0.5, 1, 2, 5, 10, 20, 50, 100, 200, and 500 nM). Then the mixtures were incubated at 37°C and fluorescence measurements of all samples were carried out on FL-2500 fluorometer. Fluorescence emission spectra of miR21-MB with miR21 are shown in Figure 4(a). A standard curve of miR21 was drawn by displaying relative fluorescence intensities (F-F0) at 578 nm and their correlation was analyzed with linear regression. The relative fluorescence intensities (F-F0) were linearly proportional to the concentration of miR21 in the range from 1 to 50 nM (Figure 4(b)). The regression equation is Y = 0.8057X + 0.2516. The regression coefficient is 0.9915. The limit of detection was lower to 0.5 nM (estimated from three times the standard deviation in the blank solution). The detection sensitivity was similar to the reported method [26, 27], but more simple with less reagents and steps.

### 3.5. Detection of miRNA-21 in Liver Cancer Cell by Molecular Beacon

**Figure 5:**
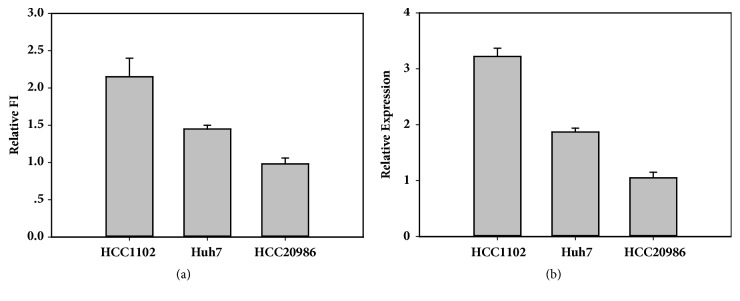
Detection of miRNA-21 in liver cancer cell by molecular beacon (a) and qRT-PCR (b).

To demonstrate that molecular beacons have the sensitivity and specificity to detect mature miRNA in a heterogeneous RNA sample, we mixed 100 nM MiR21-MB with 3 *μ*L total RNA that had been isolated from cancer cells (Huh7, HCC1102, and HCC20986). Then the mixtures were incubated at 37°C and fluorescence measurements of all samples were carried out on FL-2500 fluorometer. According to the fluorescence signal of 578 nm, the concentration of miRNA-21 was calculated by the standard curve, and the relative expression level was calculated. The expression of miRNA-21 in HCC1102 cells was higher, and the expression of HCC20986 was the lowest in Huh7 cells (Figure 5(a)). qRT-PCR is the standard technique to assess miRNA expression. The results of the two methods are similar (Figure 5(b)) indicating that the MB method will be used in bioassay.

### 3.6. Detection of miRNA-21 in Gastric Cancer Tissue by Molecular Beacon

**Figure 6:**
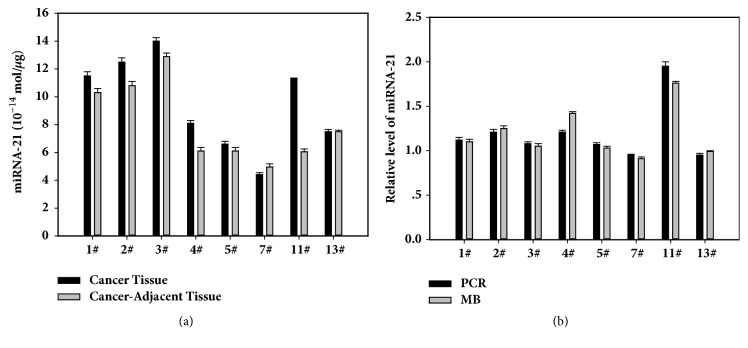
Detection of miRNA-21 in gastric cancer tissue by molecular beacon (a) and qRT-PCR (b).

We mixed 100 nM miR21-MB with 3 *μ*g total RNA that had been isolated from gastric carcinoma. Then the mixtures were incubated at 37°C and fluorescence measurements of all samples were carried out on FL-2500 fluorometer. According to the fluorescence signal of 578 nm, the concentration of miRNA-21 was calculated by the standard curve (Figure 6(a)). The expression level of miRNA-21 in 6 cases of cancer tissues was higher than that in adjacent tissues and in 1 case was lower than the adjacent tissue, and the expression level of the 1 case was similar to that in the adjacent tissues. qRT-PCR was compared with the molecular beacon, and the results of the two methods are similar (Figure 6(b)). The results showed that the microRNA expression level of tumor samples could be detected quickly and accurately by using MB method. And the results indicated that the miR21 was related to the cancer development; the expression level would be the marker to diagnosis.

## 4. Conclusions

In this paper, a fast, safe, and specific miRNA-21 quantitative method was established, with its linear range of detection in the range of 1 ~ 50 nm and the detection limit of 0.5 nm. It is used to detect the changes of miRNA-21 expression in different tumor cells and gastric cancer tissues and get similar results with qRT-PCR. So, this method will provide a reliable experimental basis for the further elucidation of the role of miRNA-21 in tumorigenesis.

## Figures and Tables

**Table 1:** Oligonucleotide strands with modifications.

Name	Sequences
miR21-m3	5′-UA$\underline{A}$CU$\underline{CG}$UCA**U**ACUGAUGUUGA-3′
miR21	5′-UAGCUUAUCAGACUGAUGUUGA-3′
MiR21-MB	5′-(TMR)$\underline{CGTTCGA}$TCAACATCAGTCTGATAAGCTA$\underline{TCGAA\;\;CG}$(DAB)-3′

## Data Availability

The data used to support the findings of this study are available from the corresponding author upon request.
