# Corrigendum to “Quantitative Detection of miRNA-21 Expression in Tumor Cells and Tissues Based on Molecular Beacon”

**DOI:** 10.1155/2020/1515794

**Published:** 2020-01-22

**Authors:** Qingxin Liu, Jialong Fan, Chuang Zhou, Liqun Wang, Bin Zhao, Haibin Zhang, Bin Liu, Chunyi Tong

**Affiliations:** ^1^College of Veterinary Medicine, Nanjing Agricultural University, Nanjing, Jiangsu 210095, China; ^2^Jiangsu Vocational College of Agriculture and Forestry, Jurong, Jiangsu 212400, China; ^3^College of Biology, Hunan University, Changsha, Hunan 410082, China

In the article titled “Quantitative Detection of miRNA-21 Expression in Tumor Cells and Tissues Based on Molecular Beacon” [1], there was an error in Figure 1 as miR21-m3 should run slower than miR21 because the molecular weight of miR21-m3 is a little bigger than miR21. The corrected version of Figure 1 is given as follows.

## Figures and Tables

**Figure 1:**
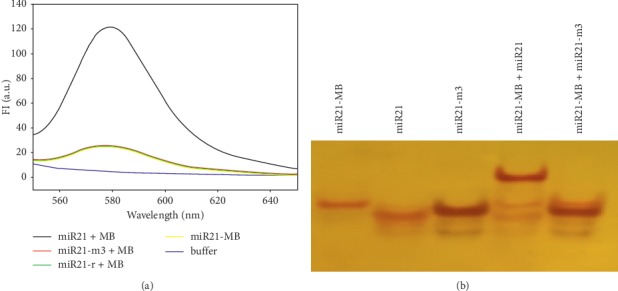
The specificity assay of MB with target by fluorescence spectrometry (a) and polyacrylamide gel electrophoresis staining by silver (b).

## References

[1] Liu Q., Fan J., Zhou C. (2018). Quantitative detection of miRNA-21 expression in tumor cells and tissues based on molecular beacon. *International Journal of Analytical Chemistry*.

