# Corrigendum #2 to “Quantitative Detection of miRNA-21 Expression in Tumor Cells and Tissues Based on Molecular Beacon”

**DOI:** 10.1155/2021/8406935

**Published:** 2021-01-13

**Authors:** Qingxin Liu, Jialong Fan, Chuang Zhou, Liqun Wang, Bin Zhao, Haibin Zhang, Bin Liu, Chunyi Tong

**Affiliations:** ^1^College of Veterinary Medicine, Nanjing Agricultural University, Nanjing, Jiangsu 210095, China; ^2^Jiangsu Vocational College of Agriculture and Forestry, Jurong, Jiangsu 212400, China; ^3^College of Biology, Hunan University, Changsha, Hunan 410082, China

In the article titled “Quantitative Detection of miRNA-21 Expression in Tumor Cells and Tissues Based on Molecular Beacon” [1], the authors' previous similar work on pre-miRNA-21 was not cited and discussed [2]. The authors said the two studies are similar in strategy, using a fluorescent probe to detect RNA, but with different probes and targets. With the structural differences of pre-miRNA and miRNA, they used a chimeric molecular beacon (the stem contains RNA bases) to detect pre-miRNA-21 in the earlier study [2], while they used a molecular beacon (entirely DNA bases) to directly detect miRNA-21 in the second article [1]. The studies were conducted at the same time and have similar outcomes, in that both LOD are 0.5 nM of the targets and there is a similar linear range (1–100 nM vs. 1–50 nM). The original version of Figure 3 that was replaced in an earlier corrigendum [3] was reproduced in error from Figure 2 in that earlier article.
